# Coarse-to-Fine Curriculum Transfer Learning Using RF-Derived Ultrasound Representations for Small-Data Breast Tumor Detection

**DOI:** 10.3390/bioengineering13070769

**Published:** 2026-06-30

**Authors:** Yu Hyun Park, Ki-Baek Lee, Hyungsuk Kim

**Affiliations:** Department of Electrical Engineering, Kwangwoon University, Seoul 01897, Republic of Korea; parkyh2024@kw.ac.kr

**Keywords:** breast ultrasound, object detection, YOLO11n, curriculum transfer learning, RF-derived ultrasound representations, deep learning

## Abstract

Breast ultrasound (BUS) is important for breast tumor detection, but speckle noise, low contrast, operator dependency, and limited medical datasets hinder robust deep learning. Although raw radiofrequency (RF) signals contain richer acoustic information than conventional B-mode images, multimodal fusion approaches often increase computational cost. To address these issues, this study proposes a curriculum transfer learning-based approach that sequentially exploits different ultrasound information representations during training. The proposed approach maintains a single detection model architecture rather than relying on complex multimodal input fusion. Phase, Envelope, and B-mode images generated from raw RF signals were defined as distinct input domains, and various training orders were evaluated. In addition, lightweight detection models based on YOLOv5, YOLOv8, YOLO11, and YOLO26 were compared to select the optimal model. A total of nine experimental settings, including single-modality training and curriculum learning conditions, were repeatedly evaluated using 100 random seeds. The experimental results showed that the proposed Phase-Envelope-B-mode (P-E-B) curriculum transfer learning strategy achieved the highest average mAP@50 among the evaluated training scenarios, with an approximately 2.08% relative improvement over single B-mode training under the fixed patient-level split and 100-seed repeated evaluation setting. The average convergence epoch was also lower than that of single B-mode training, indicating that the proposed strategy provided a favorable convergence profile while improving average validation performance. These results should be interpreted as proof-of-concept evidence obtained under a fixed patient-level split and 100-seed repeated evaluation setting, rather than as conclusive evidence of external clinical generalizability. Within this controlled small-data setting, the findings suggest that RF-derived representations may provide useful training-stage curriculum information for B-mode-based breast tumor detection while maintaining a single B-mode inference pathway.

## 1. Introduction

Breast cancer is one of the most prevalent cancers among women worldwide, and early diagnosis plays a crucial role in improving survival rates [1]. Breast ultrasound (BUS) is widely used as a diagnostic tool alongside X-ray mammography because it is non-ionizing, non-invasive, and capable of real-time imaging. In addition, BUS is highly accessible and cost-effective, making it broadly applicable in clinical practice as a primary screening and auxiliary diagnostic tool [2]. However, ultrasound images often suffer from speckle noise caused by back-scattering, low contrast, and operator dependency, which can obscure the boundaries between lesions and normal tissue. In particular, the appearance and location of the same lesion may vary depending on the probe pressure, scanning angle, and patient posture. These factors reduce image consistency and make it difficult for deep learning models to learn stable and generalizable features [2,3].

These limitations become more pronounced in deep learning-based breast tumor detection. Medical imaging datasets are generally limited in scale because data acquisition and annotation are costly, and the dataset used in this study also represents a small-data setting consisting of 200 RF scans. Under such conditions, deep learning models are prone to overfitting, as they may become excessively adapted to the training data [4]. Furthermore, most previous studies have used only B-mode images as input, although the raw radiofrequency (RF) signals contain richer acoustic information that may be partially lost during image conversion [5,6,7]. To address this limitation, multimodal fusion methods incorporating additional modalities, such as elastography and Doppler imaging, have been proposed. However, these approaches require multiple inputs to be processed simultaneously, thereby increasing model complexity and computational cost. This limits their applicability in clinical environments that require real-time detection [8,9].

To overcome these challenges, this study first compares lightweight YOLO-based models, including YOLOv5, YOLOv8, YOLO11, and YOLO26, to select a detection model suitable for the dataset used in this study. This study then proposes a curriculum transfer learning strategy that sequentially exploits different information representations during training. The proposed strategy maintains a single detection model architecture instead of employing a complex multimodal fusion structure [10]. Specifically, Phase, Envelope, and B-mode images generated from raw RF signals are regarded as distinct input domains and are learned in a stepwise manner according to predefined training orders. Subsequently, single-modality training and curriculum training with different modality orders are systematically compared [11,12,13,14]. Rather than simply reporting the best performance achieved under a specific scenario, this study aims to analyze how the learning order of different modalities affects final detection performance and training stability through repeated experiments. This study investigates the average performance changes observed in a small-scale breast ultrasound detection setting when different RF-derived information representations are sequentially exploited during training.

The novelty of this study is summarized as follows. First, unlike conventional B-mode-only detection studies, this study uses raw RF signals to derive multiple information representations, namely Phase, Envelope, and B-mode images, and examines their role as sequential training domains. Second, unlike multimodal fusion approaches that require multiple modality inputs during inference, the proposed method uses RF-derived representations only during the training stage and preserves a single B-mode inference pathway. Third, rather than evaluating only one predefined curriculum order, this study systematically compares all single-modality and curriculum-learning scenarios to examine how the order of RF-derived representations affects final detection performance and convergence behavior. These aspects position the proposed method as a training-stage curriculum transfer learning strategy for small-data breast ultrasound detection, rather than as a conventional multimodal fusion framework.

## 2. Related Works

### 2.1. Object Detection in Breast Ultrasound

Early computer-aided diagnosis (CAD) studies based on breast ultrasound primarily focused on lesion segmentation and the extraction of morphological features. In early BUS-based CAD approaches, level-set-based lesion segmentation was combined with morphological features and support vector machines (SVMs) for benign–malignant classification [15]. This type of approach represents a classical CAD pipeline before the widespread adoption of deep learning, in which lesion boundaries and shapes were quantified to improve diagnostic performance. Subsequently, fully automatic BUS segmentation methods combining spatial and frequency-domain information and boundary-regularized encoder–decoder models for anatomical structure segmentation in automated whole-breast ultrasound (AWBUS) were proposed [16,17].

With the introduction of deep learning, the research focus shifted from handcrafted feature-based CAD to convolutional neural network (CNN)-based end-to-end learning. In medical imaging in general, CNN-based end-to-end learning has been recognized as a major trend that replaces conventional handcrafted feature pipelines, and a similar transition has also been reported in BUS lesion recognition [4,5]. However, early deep learning-based detection studies largely relied on two-stage detectors, such as Faster R-CNN. Although these architectures can achieve high accuracy by separating region proposal and classification, they have limitations in terms of inference speed, which can hinder their use in real-time clinical applications [18]. Accordingly, since the introduction of YOLO and SSD, one-stage detectors have become a major approach for real-time detection by jointly considering accuracy and inference speed [19,20]. Recent studies have increasingly focused not only on accuracy, but also on real-time performance, lightweight design, and ease of deployment.

From a quantitative perspective, lightweight YOLO-based object detection models have shown continuous improvements in official COCO benchmarks [11,12,13,14,21]. According to the official Ultralytics COCO benchmark, YOLO11n achieves an mAPval 50–95 of 39.5, whereas YOLO26n reports an mAPval 50–95 of 40.9 [13,14]. In addition, the latest YOLO26n reports an mAP of 40.9 and can provide up to 43% faster CPU inference through its end-to-end NMS-free design [14]. These results indicate that recent real-time detectors have improved not only in detection accuracy but also in lightweightness, latency, and deployment efficiency. However, these official results are based on general object detection datasets and cannot be directly assumed to transfer to breast ultrasound environments, where speckle noise is severe and the amount of available data is limited. Therefore, in practical BUS applications, it is necessary to separately verify the stability and resistance to overfitting of such models in small-scale medical data settings, rather than relying solely on their superiority in general benchmarks.

Recently, a two-stage detection–segmentation framework combining YOLOv5-based mass ROI detection with GOLO-CMSS-based lesion segmentation has been proposed [22]. This indicates that recent studies have expanded beyond simple lesion classification toward complex pipelines that combine detection and segmentation while utilizing multi-scale information and global–local contextual cues. However, such approaches are structurally complex and may increase both training and inference costs because detection and segmentation must be performed sequentially. Therefore, in environments where limited data and real-time performance must be considered simultaneously, strengthening the representation learning capability of a single detector, rather than adding a complex downstream segmentation branch, can also be considered a meaningful research direction.

Despite these advances, several challenges remain in breast ultrasound imaging, including speckle noise, low contrast, operator dependency, and small-data limitations. In particular, achieving stable performance in limited-data settings without relying on complex multi-branch architectures remains an insufficiently resolved problem. In this context, the present study can be positioned as an attempt to improve both performance and efficiency by introducing modality-wise sequential learning from the perspective of training strategy, while using a recent lightweight detector as the base architecture.

### 2.2. Multimodal Learning and Curriculum Learning

To overcome the information loss associated with single B-mode images, several multimodal fusion models combining elastography or Doppler imaging have been proposed [8,9]. These studies improved detection accuracy by incorporating additional information, such as lesion stiffness or blood flow. However, because two or more modalities must be processed simultaneously, these methods substantially increase model complexity and computational cost. This increased burden is a major factor that limits real-time applicability in low-resource hardware environments, such as point-of-care ultrasound (POCUS) systems.

As an efficient alternative, curriculum learning has attracted attention as a strategy that facilitates model convergence by controlling the training order according to data difficulty or information content [10]. In the field of medical imaging, several approaches have been explored, such as training models first on less noisy data or gradually narrowing the learning scope from full images to local patches [23,24].

In this study, rather than employing a complex fusion model, we propose a coarse-to-fine curriculum strategy that sequentially exploits Phase and Envelope information derived from RF signals during training. This approach differs from conventional multimodal fusion studies in that it maintains a single B-mode input structure at the inference stage while sequentially utilizing different RF-derived representations during the training process.

## 3. Materials and Methods

### 3.1. Dataset and RF-Derived Representations

**Figure 1:**
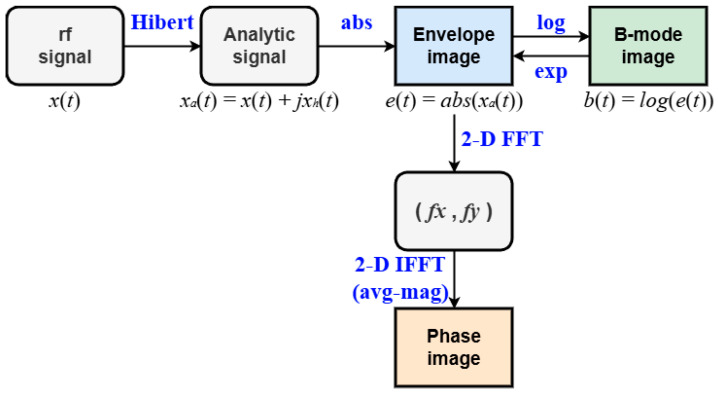
RF signal processing framework for generating Envelope, B-mode, and Phase images. The analytic signal was obtained using the Hilbert transform, followed by envelope extraction, log-compressed B-mode conversion, and phase image reconstruction based on frequency-domain processing [7].

This study used the publicly available breast ultrasound dataset OASBUD. OASBUD consists of 100 breast lesions acquired from 78 women, including 52 malignant and 48 benign lesions, and two orthogonal scans are provided for each lesion [25]. The original RF echo data in OASBUD were acquired at the Institute of Oncology, Warsaw, Poland, using an Ultrasonix SonixTouch Research ultrasound scanner with an L14-5/38 linear array transducer (Ultrasonix Medical Corporation, Richmond, BC, Canada) [25]. Therefore, the raw RF scan data used in this study comprised a total of 200 scans, corresponding to 104 malignant and 96 benign scans. In this study, signal processing was applied to the raw RF signal of each scan to generate three modality images, namely Envelope, B-mode, and Phase images, which were subsequently used for model training. Figure 1 illustrates the overall signal processing pipeline used to generate Envelope, B-mode, and Phase images from raw RF signals.

The Envelope image was generated from the magnitude component of the analytic signal obtained by applying the Hilbert transform to the RF signal [7]. As a representation that preserves information before log compression, the Envelope image retains tissue-specific echogenicity in a relatively linear manner. Therefore, Envelope images may be advantageous for learning fine tissue scattering characteristics or signal intensity distributions and can provide complementary raw signal-level information that may be partially lost in B-mode images.

The B-mode image is a conventional ultrasound representation generated by applying log compression to the Envelope image and is the most widely used image format in clinical settings [7]. Because its brightness contrast is adjusted to facilitate human visual interpretation, B-mode imaging is useful for intuitively identifying the overall morphology and intensity distribution of lesions. However, during log compression, some detailed information contained in the raw signal may be compressed or distorted, which may limit the preservation of subtle tissue characteristics.

The Phase image is generated based on phase information reconstructed in the frequency domain and is a representation that emphasizes instantaneous changes or discontinuities rather than absolute signal intensity [7,26]. Owing to these characteristics, Phase images can highlight geometric cues such as lesion boundaries, structural transitions, and anatomical morphology, rather than the intensity distribution within tissues itself. Consequently, Phase images provide structure-oriented information that differs from B-mode and Envelope images and may play a meaningful role in learning the morphological characteristics of tumors.

**Figure 2:**
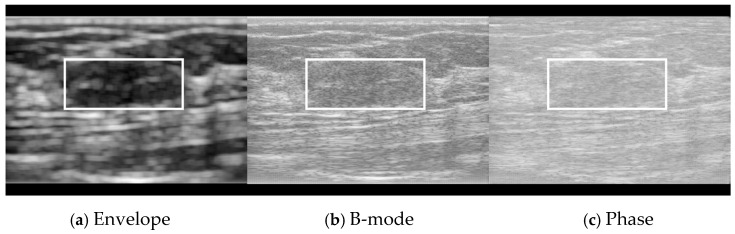
Representative Envelope, B-mode, and Phase images generated from the same OASBUD lesion. The white box indicates the lesion region of interest [25].

Figure 2 presents representative examples of the three modalities generated from the same lesion, namely Envelope, B-mode, and Phase images.

Although the three modalities used in this study were derived from the same raw RF signal, they represent different information characteristics and provide distinct levels of visual and physical features. Therefore, this study sequentially utilized these modalities during training to expand information diversity in a limited medical data setting and to compensate for the limitations of single-modality learning. This perspective constitutes one of the key assumptions underlying the proposed curriculum transfer learning strategy.

### 3.2. Data Splitting Strategy

In deep learning studies using small-scale medical datasets, preventing performance overestimation caused by data leakage is critically important [27,28]. In ultrasound imaging, multiple slices or scans can be acquired from the same patient. Therefore, if image-level splitting is applied, information from the same patient may be included in both the training and validation sets [27,28]. To prevent this issue, this study used patient ID, rather than individual images, as the basic unit of data splitting. This ensured that image information from a given patient was not duplicated across different subsets.

In addition, considering that the overall dataset maintained a relatively balanced class distribution between benign and malignant lesions, stratified sampling was applied so that the original class ratio was preserved as much as possible in each split. This procedure was intended to prevent excessive class imbalance in a specific subset and to avoid distortion of validation performance caused by random sample composition. Consequently, the splitting strategy used in this study was designed to maintain fairness and statistical consistency in the evaluation, even under limited-data conditions.

To ensure fair comparisons across model architectures and curriculum orders, the same patient-level train/validation split was fixed and used in all experiments [27,28]. Random seeds 101–200 were used not for data splitting, but to repeatedly evaluate stochastic factors in the training process, such as model initialization, data augmentation, and mini-batch ordering. In other words, by maintaining the same split condition throughout the YOLO architecture comparison experiments and subsequent curriculum transfer learning experiments, we controlled the experimental setting so that performance differences could be attributed to model architecture and training strategy rather than random data sampling.

This design enables a more consistent comparison of repeated experimental performance distributions while controlling for variation caused by data splitting. In particular, this study fixed the same patient-level split instead of generating a new train/validation split for each seed, thereby minimizing the influence of split variance on comparisons among model architectures or curriculum orders. Therefore, the 100-random-seed repeated experiments in this study should be interpreted as controlled experiments designed to compare performance stability against stochasticity in the training process, such as initialization, data augmentation, and mini-batch ordering, under the same patient-level split condition, rather than as an evaluation of generalization performance across different data splits. However, this setting does not replace validation using an independent test set or an external dataset. The purpose of this study is to compare relative trends among training strategies in a limited RF ultrasound dataset, and external generalization performance should be further validated in future studies.

### 3.3. Model Architecture Selection

To compare the performance of object detection models as fairly as possible, all experiments in this study were conducted within a single Ultralytics framework [11,12,13,14]. This was done because using different implementations may introduce differences in the training pipeline, including data augmentation, loss functions, and learning schedules, which can affect performance comparisons. Therefore, by maintaining the same library environment and the same data split condition, the experiments were designed so that the observed performance differences would be more closely related to model architecture than to implementation-level variation.

The comparison models were selected to reflect the technical evolution of the YOLO family and included YOLOv5, YOLOv8, YOLO11, and YOLO26. YOLOv5 was used as a baseline representing an anchor-based detection approach, whereas YOLOv8 was included to examine the effect of an anchor-free architecture. YOLO11 was considered as a candidate model incorporating recent lightweight architectural improvements and attention-based feature enhancement components. YOLO26 was included to examine whether an end-to-end NMS-free detection structure is also effective in a small-scale medical imaging environment [11,12,13,14]. This configuration was intended to compare how the evolution of object detection architectures affects performance in a domain-specific setting such as breast ultrasound imaging.

### 3.4. Curriculum Transfer Learning Strategy

Based on the backbone comparison experiment, this study selected YOLO11n as the base model and designed a sequential transfer learning framework that reflects differences in the level of abstraction among modality-specific information representations [10,29]. The central hypothesis of this strategy is that, rather than randomly mixing ultrasound modalities with different physical characteristics, gradually learning from relatively abstract representations to more concrete representations may allow the model to sequentially experience structural lesion cues and signal intensity information, leading to lower initial loss and improved average performance during the final B-mode training stage [10,29]. From this perspective, this study regarded Phase, Envelope, and B-mode images generated from RF signals as independent input domains and systematically evaluated the effect of their learning order on final detection performance.

To this end, a total of nine training scenarios were constructed. First, three single-modality scenarios using B-mode, Envelope, and Phase images were defined to examine the individual learning effect of each modality. Subsequently, six curriculum orders were added to include all possible permutations of the three modalities and to evaluate the effect of modality learning order. This configuration allows the proposed curriculum strategy to be assessed not only in terms of its own performance, but also in terms of which modality orders tend to yield relatively favorable average performance and which orders may be associated with performance degradation under the same experimental framework. In particular, this study focuses on the coarse-to-fine hypothesis, namely whether a learning order that begins with structure-oriented abstract information and gradually proceeds toward visually concrete information shows a relatively favorable average performance trend in medical ultrasound detection.

**Figure 3:**
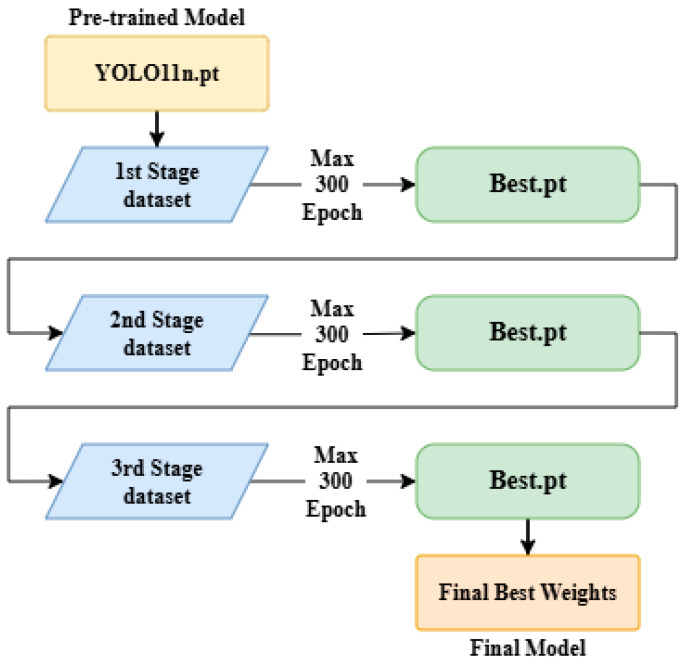
Curriculum transfer learning framework. The best checkpoint from each modality stage is transferred as the initial weight for the next stage.

In each curriculum order, stage-wise training was connected through weight transfer. Specifically, after each training stage was completed, the model weights corresponding to the lowest validation loss were saved as best.pt and used as the initialization parameters for the next training stage. This design allowed the weights learned in the preceding stage to be used as the initial parameters of the subsequent stage. In other words, the proposed curriculum strategy was implemented not merely as a change in the order of data presentation, but as a transfer learning structure in which the weights obtained from each modality-specific training stage were passed to the next stage as initial parameters. Figure 3 schematically illustrates this stage-wise weight transfer process.

Because this study required large-scale repeated experiments across nine training conditions, an implementation strategy was needed to reduce computational cost while maintaining experimental consistency. Therefore, an individual cache strategy was introduced, in which the best.pt weights obtained from preceding stages were saved separately and reused in subsequent experiments. This strategy made it possible to directly start later-stage experiments without repeatedly training identical preceding stages, thereby preventing redundant training and substantially reducing the overall experimental time. Thus, the proposed framework was designed to evaluate the effect of modality-based curriculum learning from a methodological perspective while also ensuring computational efficiency sufficient for large-scale repeated experiments.

### 3.5. Experimental Setup

The experimental setup of this study focused on quantitatively evaluating the standard deviation of results that frequently occurs in small-scale medical datasets and minimizing accidental performance variation caused by specific initialization conditions. The entire dataset was divided into training and validation sets at a ratio of 8:2. To avoid the possibility of cherry-picking, in which unusually high performance is observed from a single seed, 100 independent random seeds ranging from 101 to 200 were fixed and used for repeated experiments.

Accordingly, each training scenario was repeatedly validated under the same procedure, resulting in a total of 900 scenario-level evaluations, corresponding to nine scenarios multiplied by 100 random seeds. Therefore, the performance reported in this study reflects distributional trends obtained through repeated experiments rather than the result of a single run. This design was intended to compare the average performance tendency of each training scenario under stochastic variations in initialization and training processes, without relying on accidental high-performance results from a specific seed.

All experiments were conducted using Python 3.10.12 and Ultralytics 8.4.59 with PyTorch 2.12.0+cu130 and torchvision 0.27.0+cu130, and the same training settings were applied to all scenarios for fair comparison. Specifically, the maximum number of training epochs was set to 300, the early stopping patience was set to 50, the input image size was fixed at 640, and the batch size was set to 16. In addition, the entire experimental process was controlled within a single framework to ensure that performance differences among experiments did not originate from implementation-level variation. During data splitting, patient-level stratified splitting was applied to prevent data leakage, ensuring that information from the same patient was not included in both the training and validation sets [27,28]. These settings provide an experimental basis for more objective comparisons of model architectures and curriculum strategies.

To improve reproducibility, additional implementation details are summarized in Table 1. The train/validation split was implemented using text files that listed the image paths assigned to each subset, rather than by physically moving images into separate train and validation folders. For each random seed, modality-specific train and validation text files were generated for B-mode, Envelope, and Phase images. In the representative split used for seed 101, each modality contained 160 training image-list entries and 40 validation image-list entries. The corresponding class-label distribution was 77 benign and 83 malignant entries in the training set, and 19 benign and 21 malignant entries in the validation set. The same training configuration, augmentation policy, and evaluation settings were applied consistently across the model-comparison and curriculum-learning experiments. Because the train/validation split was performed at the patient-ID level, no patient appeared in both subsets. However, the object-detection framework used RF scan-derived image files as the actual training input units. Therefore, the split statistics are reported using the image-list entries consumed by the Ultralytics training pipeline, together with their benign/malignant class-label distributions.

## 4. Results

### 4.1. Benchmark of Lightweight YOLO Backbones

Before applying curriculum learning, a benchmark experiment was conducted to select the optimal backbone model, and the results are presented in Table 2. This experiment was performed using the same data split condition while changing only the detection model, and each model was evaluated over 100 random seeds.

To validate the object detection performance of the proposed framework, comparative experiments were conducted using recent lightweight models from the YOLO series. Among the n-scale models, YOLO11n achieved the highest average detection performance, with an mAP@50 of 0.7403, Recall of 0.6786, and Precision of 0.7727. In addition, its performance was comparable to that of the s-scale models while maintaining a smaller model size and lower computational burden, making it suitable as the base model for subsequent repeated experiments.

In this experiment, YOLO11n was identified as the most balanced candidate in terms of lightweight design, average detection performance, and feasibility for repeated experiments. However, because the internal activation patterns or attention maps of C2PSA were not separately analyzed in this study, the superior performance of YOLO11n was interpreted based on experimental results rather than attributed to a specific architectural mechanism. In contrast, YOLO26n achieved an mAP@50 of 0.6738 in this experiment, which was lower than that of YOLO11n. Therefore, considering model lightweightness, average detection performance, and the feasibility of repeated experiments across the subsequent nine curriculum scenarios, YOLO11n was selected as the final backbone model.

### 4.2. Analysis of Box Loss Curves

**Figure 4:**
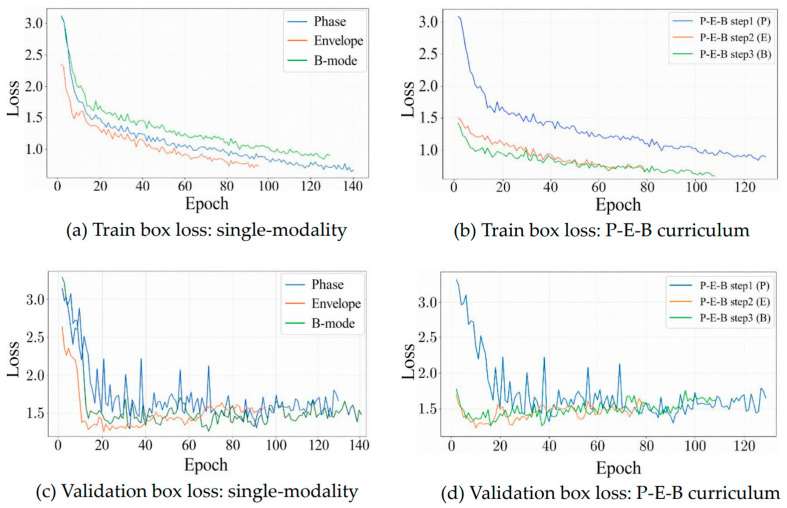
Training and validation box losses under seed 101. (**a**) Training box loss for single-modality learning; (**b**) training box loss for the P-E-B curriculum; (**c**) validation box loss for single-modality learning; and (**d**) validation box loss for the P-E-B curriculum.

Figure 4 shows the changes in training and validation box loss observed for single-modality learning and the proposed P-E-B curriculum learning under seed 101 among the 100 repeated experiments. Seed 101 was not selected because it produced the highest performance, but was used as the first fixed seed to provide an illustrative example of learning dynamics. Therefore, this figure should be interpreted as a representative example of the training process and was not used as direct evidence for quantitative performance superiority. The final performance comparison and methodological interpretation were based on the 100-seed repeated experimental results presented in Section 4.3.

Figure 4a shows the training box loss when Phase, Envelope, and B-mode were each trained independently. In all three single-modality conditions, the training box loss generally decreased as the training epochs progressed. Each modality also terminated training at a different epoch, which was caused by the early stopping criterion applied in this study when further meaningful improvement was no longer observed. Thus, although overall convergence was observed in single-modality learning, differences existed in convergence speed and termination point depending on the modality.

Figure 4b shows the stage-wise training box loss of the proposed P-E-B curriculum learning strategy. In Step 1, Phase training began with a relatively high loss and gradually decreased as training progressed. In Step 2, Envelope training was initialized using the checkpoint from Step 1, and therefore began from a lower loss level than Step 1 while continuing to decrease through additional training. Finally, in Step 3, B-mode training started from an even lower initial loss than Step 2 and converged to the lowest training box loss among the three stages. In the representative seed 101 example, the P-E-B curriculum started each subsequent stage with a lower initial training box loss and converged to the lowest loss level in the final B-mode stage.

Figure 4c,d show the validation box loss for single-modality learning and P-E-B curriculum learning, respectively. In both cases, the validation loss decreased during the early training phase and then fluctuated or partially increased in the later phase. This indicates that, under the experimental conditions of this study, the validation loss tended to saturate or partially increase in later training epochs. Therefore, early stopping was applied to prevent unnecessary additional training after validation performance had saturated, and the checkpoint with the most appropriate validation performance was used as the final model.

In particular, comparison between single B-mode learning in Figure 4a and the final Step 3 B-mode learning of the P-E-B curriculum in Figure 4b indicates that the loss dynamics can differ depending on the training path, even when the same B-mode input is used. Single B-mode learning started from a high initial training loss similar to that of Phase and terminated with a relatively high final loss among the three single modalities. In contrast, the final B-mode stage of the P-E-B curriculum started from a lower initial loss based on the weights transferred from the preceding Phase and Envelope stages and eventually reached a lower final loss level.

In the learning curve of seed 101, the final B-mode stage of the P-E-B curriculum showed both lower initial loss and lower final loss than single B-mode learning. Therefore, Figure 4 was interpreted as a representative example showing that the loss dynamics changed after stage-wise weight transfer under the P-E-B condition.

### 4.3. Curriculum Learning Evaluation

The average performance and repeated-experiment distribution tendencies of the nine training scenarios were compared using 100 random seeds. The results showed that the P-E-B curriculum, corresponding to the Phase—Envelope—B-mode order, achieved the highest mean values for mAP@50, Precision, and Recall. However, because the differences from other high-performing curriculum orders, such as E-P-B, P-B-E, and B-P-E, were limited, these results should not be interpreted as definitive evidence of the absolute superiority of P-E-B. Rather, they indicate that the coarse-to-fine learning order produced the most favorable average performance profile among the evaluated training scenarios in the 100-seed repeated experiments. This comparison evaluated three single-modality learning conditions and six curriculum orders under the same experimental conditions, and the scenarios were comprehensively analyzed in terms of mAP@50, Precision, Recall, and convergence epoch. The complete quantitative results are presented in Table 3.

As shown in Table 3, the P-E-B curriculum achieved the highest average values among the nine training conditions in terms of mAP@50, Precision, and Recall. However, because the standard deviations were not negligible and the mean differences among the top-performing scenarios were limited, these results were interpreted as a relatively favorable average performance tendency observed in repeated experiments, rather than as conclusive evidence of statistical superiority.

To further examine whether the observed differences were statistically supported, seed-wise paired statistical comparisons were performed between P-E-B and selected training scenarios. Paired *t*-tests, Wilcoxon signed-rank tests, and 95% bootstrap confidence intervals were used for mAP@50 and Recall. As shown in Table 4, P-E-B showed statistically supported improvements over single B-mode training in both mAP@50 and Recall. However, the differences between P-E-B and the other high-performing curriculum orders were not consistently statistically significant. Therefore, P-E-B should be interpreted as providing a statistically supported improvement over single B-mode training, while its advantage over other curriculum-learning alternatives should be considered a favorable average tendency rather than conclusive statistical superiority.

Single B-mode learning achieved an mAP@50 of 0.7293 ± 0.0622 and a Recall of 0.6722 ± 0.0692, whereas P-E-B produced higher average values. In contrast, the B-E-P scenario showed an mAP@50 of 0.6562 ± 0.0635 and a Recall of 0.5926 ± 0.0740, indicating lower average performance than single B-mode learning. These results demonstrate that, even when the same modality set is used, the learning order can meaningfully influence the final average detection performance.

**Figure 5:**
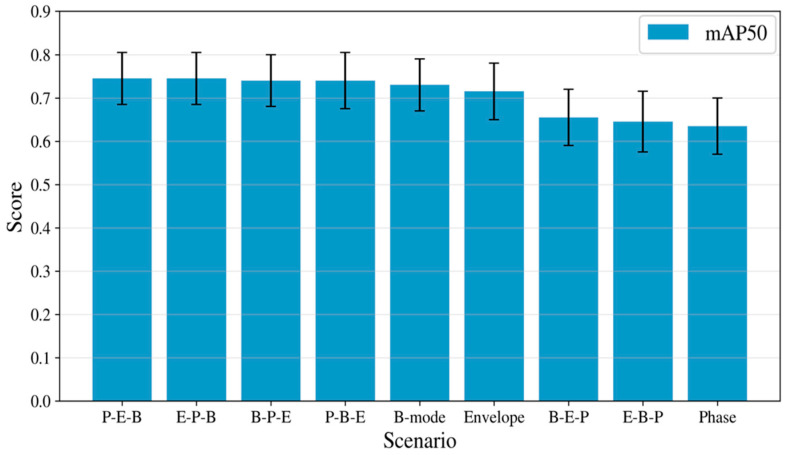
Scenario-wise comparison of mean mAP@50 with standard deviation across 100 seeds.

This tendency is also observed in Figure 5, which presents the scenario-wise mean mAP@50 and standard deviation based on the 100-seed repeated experiments. In Figure 5, the P-E-B curriculum was positioned among the highest-performing scenarios in terms of the mean value, and a similar tendency was observed in the repeated-experiment distribution. However, because the differences from E-P-B and P-B-E were limited, it is more appropriate to summarize the results as showing relatively high average performance for coarse-to-fine learning orders, rather than claiming the absolute superiority of a specific scenario. This interpretation is based on the mean and standard deviation across 100 random seeds, rather than on a single experimental run.

The means and standard deviations of Precision and Recall for each scenario are also presented in Table 3. The P-E-B curriculum showed high average performance in both metrics. Differences among scenarios were also observed in terms of learning efficiency. The single B-mode model converged at an average of 136.73 ± 48.27 epochs, whereas the P-E-B curriculum reached its optimal performance at an average of 116.00 ± 46.79 epochs. In addition, B-P-E and P-B-E also showed relatively fast convergence tendencies, with 104.62 ± 45.34 and 103.03 ± 40.14 epochs, respectively. Therefore, under the experimental conditions of this study, some curriculum orders tended to reach their optimal performance with fewer epochs than single-modality learning.

**Figure 6:**
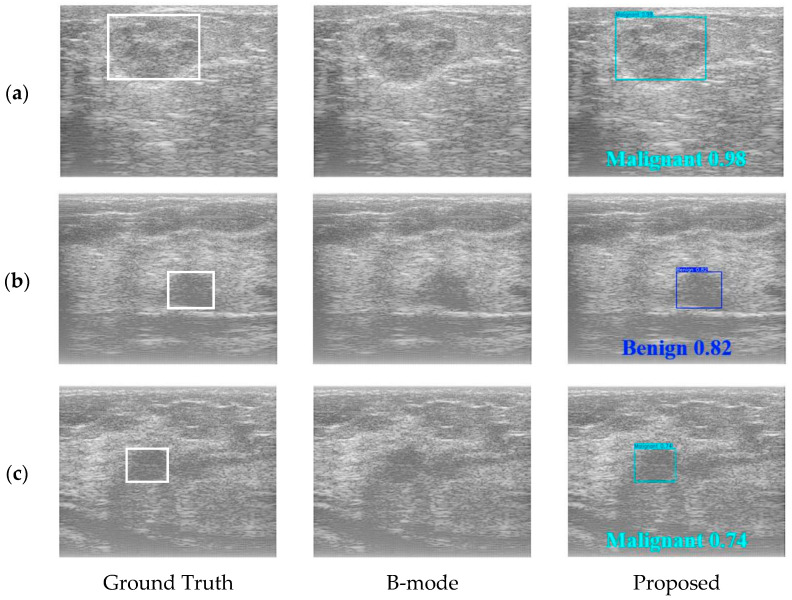
Qualitative comparison of lesion detection results for validation B-mode images missed by the single B-mode baseline model. (**a**–**c**) Three representative baseline-missed cases. The columns show the expert-annotated ground truth, the single B-mode baseline output, and the proposed P-E-B curriculum model output, respectively. Bounding boxes indicate lesion regions; boxes in the ground-truth column denote expert annotations, whereas boxes in the model-output columns denote predicted lesion locations. In the proposed-model column, the enlarged class-confidence text below each image reproduces the original YOLO detection label for readability. Absence of a bounding box indicates no detection.

Figure 6 presents qualitative illustrative examples selected from validation B-mode images missed by the single B-mode baseline model, comparing expert-annotated ground-truth lesion boxes, baseline model outputs, and proposed P-E-B curriculum model outputs. For readability, the class-confidence label of each proposed-model prediction is also displayed in enlarged form below the corresponding image. Each row shows the same validation B-mode image under three visualization conditions: the first column shows the ground-truth lesion annotation, the second column shows the result of the single B-mode baseline model, and the third column shows the result of the proposed P-E-B curriculum model. These examples were selected to visualize cases in which the baseline and proposed models showed different detection behavior. In the presented cases, the baseline model failed to detect the lesion or localized it insufficiently, whereas the proposed model detected lesion regions closer to the expert-annotated ground-truth boxes. However, these qualitative examples should be interpreted only as illustrative cases and not as representative of all validation samples or as general evidence of overall performance. The overall performance tendency should be evaluated based on the quantitative results in Table 3 and Figure 5.

## 5. Discussion

### 5.1. Performance-Based Interpretation of the Coarse-to-Fine Tendency

In the OASBUD-based breast ultrasound detection experiments conducted in this study, the P-E-B learning order, which corresponds to the Phase–Envelope–B-mode sequence, achieved the highest average values for mAP@50, Precision, and Recall among the nine training conditions. Compared with single B-mode training, the P-E-B curriculum showed higher average mAP@50 and Recall, while also showing a tendency toward fewer convergence epochs. These results indicate that sequentially learning RF-derived information representations can improve the average detection performance of a B-mode-based detector under the experimental conditions of this study.

However, because the differences among the top-performing curriculum orders were limited, these results should not be interpreted as evidence of the absolute superiority of the P-E-B order. Rather, they indicate that coarse-to-fine learning orders showed relatively favorable average performance under the fixed patient-level split and 100-seed repeated evaluation setting. In contrast, performance degradation was observed in some curriculum orders, such as B-E-P, suggesting that the final average performance can vary depending on the learning order, even when the same modalities are used, which is consistent with the broader transfer-learning literature describing possible negative transfer effects [30].

This tendency can be interpreted from the perspective of modality-specific information representations. Phase images emphasize changes and discontinuities rather than absolute signal intensity and can provide boundary-related and structural information. Envelope images preserve reflection-intensity information before log compression and can provide signal-level information that may be partially compressed in B-mode images. B-mode images are conventional ultrasound representations generated through log compression and are the most widely used image format in clinical settings. Therefore, the P-E-B sequence can be regarded as a sequential learning condition that progresses from structure-oriented input, to reflection-intensity-based input, and finally to clinically used B-mode input.

Nevertheless, this study did not directly analyze internal feature representations, layer-wise activations, attention maps, or representation similarity across the sequential training stages. Therefore, the proposed coarse-to-fine explanation should be understood as a performance-based interpretation derived from the observed curriculum-order results and the physical characteristics of the RF-derived representations, rather than as direct mechanistic evidence from feature-level analysis. In this regard, the present findings support the empirical observation that the order of learning Phase, Envelope, and B-mode representations can influence final average detection performance, but they do not fully explain how the internal representation space changes during curriculum transfer learning. Future studies should incorporate Grad-CAM [31], feature similarity analysis, UMAP or t-SNE embedding, layer-wise activation comparison, and centered kernel alignment analysis to more directly examine whether coarse-to-fine sequential learning induces systematic changes in learned feature representations.

### 5.2. Negative Transfer in Fine-to-Coarse Ordering

In contrast to the P-E-B curriculum, the B-E-P scenario showed lower average mAP@50 and Recall than single B-mode learning. In this study, this result was not directly defined as negative transfer [30], but was conservatively interpreted as an average performance degradation observed under a fine-to-coarse learning order. This result indicates that sequential learning does not automatically improve detection performance, and that the direction of transfer among RF-derived representations can influence final average performance.

B-mode images are clinically interpretable representations generated through log compression and provide relatively intuitive lesion contrast and visual morphology. However, in the B-E-P condition, where training started from B-mode and was subsequently transferred to Envelope and Phase stages, the final average performance decreased. When learning proceeded from B-mode to Envelope and Phase, which are relatively more primitive and abstract representations, this result was interpreted as being associated with a mismatch in the fine-to-coarse transfer direction.

This interpretation is consistent with the quantitative results of this study. The B-E-P scenario showed lower mAP@50 and Recall than single B-mode learning, whereas the P-E-B order showed the highest average mAP@50, Precision, and Recall among the evaluated scenarios. These results suggest that the sequential use of modalities itself is not sufficient, and that the learning order should be considered when applying curriculum transfer learning to RF-derived ultrasound representations.

### 5.3. Clinical Feasibility

The proposed curriculum transfer learning strategy has structural simplicity because it maintains a single B-mode input structure at the inference stage. Unlike multimodal fusion approaches, which require multiple inputs to be processed simultaneously, the proposed strategy uses RF-derived representations only during the training process. Therefore, Phase and Envelope images are not required as additional inputs at the inference stage, and the final detector can maintain the conventional B-mode-based ultrasound workflow.

This characteristic is important because one of the motivations of this study was to utilize the acoustic information contained in raw RF signals without increasing the complexity of the inference-stage model structure. In this study, RF-derived Phase and Envelope images were sequentially used during training, but the final detection model remained based on B-mode input. Therefore, the proposed approach can be interpreted as a training strategy that expands information diversity during learning while preserving a simple B-mode-based inference structure.

It should be clarified that this study does not provide a direct experimental comparison with multimodal fusion architectures. Therefore, the proposed approach should not be interpreted as evidence of superiority over fusion-based methods. Rather, the objective of this study is to investigate whether RF-derived representations can be used as training-stage curriculum information while maintaining a conventional single B-mode input structure at inference. From this perspective, the proposed method is intended as a training-phase strategy for improving representation learning under limited-data conditions, not as a replacement for multimodal fusion approaches.

From a false-negative-sensitive detection perspective, Recall is an important metric in breast tumor detection because missed tumor regions may delay further diagnostic assessment. In the object-detection setting used in this study, Recall represents the proportion of annotated tumor objects that were successfully detected and can therefore be interpreted as a detection-level sensitivity-related metric. The P-E-B curriculum showed a higher average Recall than single B-mode training across repeated experiments, and the paired statistical analysis further indicated a statistically supported Recall improvement over single B-mode training. This result suggests that the proposed curriculum may help reduce missed detections under the controlled validation setting.

However, the clinical interpretation of this result should remain cautious. The present study was based on image-level object detection using a limited public RF ultrasound dataset, and it did not perform a dedicated lesion-level clinical validation. In addition, classification-style specificity and false-positive rate require a clear definition of true-negative images or non-lesion regions, which is not fully addressed by the current bounding-box detection protocol. Therefore, although the improved Recall is relevant from a false-negative-sensitive screening perspective, the present results should not be interpreted as sufficient evidence of clinical diagnostic effectiveness. Future studies should include lesion-level sensitivity, specificity, false-negative rate, false-positive rate per image, and external validation using independent clinical cohorts.

### 5.4. Limitations

This study has several limitations.

First, the experiments were conducted using only the OASBUD public RF ultrasound dataset, and validation using independent datasets acquired from external institutions or different ultrasound systems was not included. Therefore, the results should be interpreted as proof-of-concept evidence obtained in a limited dataset environment where RF signals are available. Additional validation is required before the findings can be directly generalized to broader breast ultrasound settings.

Second, this study performed repeated experiments using 100 random seeds while fixing the same patient-level train/validation split. This setting is appropriate for removing the influence of data split variation and comparing stochasticity during the training process. However, it does not replace final generalization evaluation using an independent test set. Moreover, because the reported performance was obtained from repeated experiments on the validation split, it should not be interpreted as final performance on an unseen patient cohort.

In addition, because the dataset used in this study is small, the reported performance may be sensitive to training hyperparameters, data augmentation settings, and early stopping criteria. To reduce the possibility that the results were driven by a single favorable run or by inconsistent training settings, the same augmentation policy, maximum epoch setting, early stopping patience, batch size, image size, and Ultralytics-based training framework were applied across all scenarios, and each condition was evaluated using 100 random seeds. Nevertheless, this study did not independently analyze the sensitivity of the proposed curriculum strategy to different augmentation policies or early stopping criteria. Future work should therefore include robustness analyses using multiple augmentation policies, early stopping patience values, and validation protocols to further examine the stability of the observed curriculum-learning trends.

Third, although the P-E-B curriculum achieved the highest mean values for mAP@50, Precision, and Recall, the performance differences among the top-performing curriculum orders were limited. Therefore, the results of this study should be interpreted not as evidence of the absolute superiority of a specific curriculum order, but as an indication that RF-derived modality curricula following a coarse-to-fine tendency showed relatively favorable average performance under the experimental conditions of this study.

Finally, this study did not directly analyze how sequential learning with Phase, Envelope, and B-mode images changes internal feature representations. Therefore, the coarse-to-fine interpretation is based on performance results and modality characteristics. Further validation using Grad-CAM [31], feature similarity analysis, and layer-wise activation analysis is needed. In addition, reader study performance, prospective validation, and inference time analysis should be evaluated in future studies to further assess practical feasibility.

## 6. Conclusions

In this study, we proposed a coarse-to-fine curriculum transfer learning strategy using Phase, Envelope, and B-mode modalities generated from raw RF signals to mitigate the persistent challenges of data scarcity and speckle noise in breast ultrasound object detection. The proposed approach was designed to examine whether detection performance and learning efficiency in a limited medical data setting could be improved by sequentially exploiting input representations with different physical characteristics during training, without using a complex multimodal fusion architecture. To this end, YOLO11n was selected as the final backbone through preliminary comparative experiments among YOLO-based models. Subsequently, a total of nine training scenarios, including three single-modality settings and all possible permutation-based curriculum orders, were repeatedly evaluated using 100 random seeds. The results showed that the P-E-B curriculum achieved higher average mAP@50 and Recall than single B-mode training, while also showing a tendency toward reduced convergence epochs.

However, these results should be interpreted as an average performance tendency observed under limited differences among the top-performing scenarios, rather than as definitive evidence of the absolute superiority of a specific curriculum order. In this study, coarse-to-fine sequential learning showed relatively favorable average performance under the experimental conditions. In contrast, performance degradation was observed in some scenarios, indicating that not only the sequential use of modalities itself, but also the order in which the modalities are learned, can affect the final performance tendency. These findings suggest that RF-derived modality-based curriculum transfer learning may serve as an effective training strategy for small-scale breast ultrasound detection, while further validation using independent datasets, external cohorts, and clinical evaluation protocols is required to confirm its generalizability and practical applicability.

## Figures and Tables

**Table 1:** Detailed experimental and implementation settings used in this study.

Category	Setting
Dataset	OASBUD public RF breast ultrasound dataset
Total dataset	78 women, 100 lesions, 200 RF scans
Class labels	Benign and Malignant
Split strategy	Patient-level stratified train/validation split
Split implementation	Text-file-based image path lists
Train/validation ratio	8:2
Training image-list entries	160 per modality
Validation image-list entries	40 per modality
Training class-label distribution	77 benign, 83 malignant
Validation class-label distribution	19 benign, 21 malignant
Modalities	B-mode, Envelope, and Phase
Programming language	Python 3.10.12
Framework	Ultralytics 8.4.59
Deep learning backend	PyTorch 2.12.0+cu130; torchvision 0.27.0+cu130
Numerical computing	NumPy 2.2.6; SciPy 1.15.3
Image processing	OpenCV 4.13.0
Visualization	Matplotlib 3.10.9
Input image size	640 × 640
Batch size	16
Maximum epochs	300
Early stopping patience	50
Random seeds	101–200
Optimizer	Ultralytics auto optimizer setting
Initial learning rate	0.01
Final learning rate factor	0.01
Momentum	0.937
Weight decay	0.0005
Warm-up epochs	3.0
NMS IoU threshold	0.7
Confidence threshold	Not manually overridden; the default Ultralytics validation setting was used
Patient overlap between subsets	Not allowed; split performed at the patient-ID level
Reported split unit	RF scan-derived image-list entries used by the training framework
Augmentation settings	HSV augmentation; translation = 0.1; scale = 0.5; horizontal flip = 0.5; mosaic = 1.0; mixup = 0.0; cutmix = 0.0; auto augment = randaugment; erasing = 0.4
Operating system	Ubuntu 22.04.5 LTS
CPU	AMD Ryzen 9 5950X 16-Core Processor (Advanced Micro Devices, Inc., Santa Clara, CA, USA)
RAM	128 GiB
GPU	Two NVIDIA GeForce RTX 3090 GPUs, 24 GB each (NVIDIA Corporation, Santa Clara, CA, USA)
GPU driver	560.35.03

**Table 2:** Comparative results of lightweight YOLO backbone candidates at n/s scales.

Model	mAP@50	Recall	Precision
YOLOv5n	0.7083 ± 0.0631	0.6505 ± 0.0834	0.7391 ± 0.0947
YOLOv8n	0.7009 ± 0.0602	0.6536 ± 0.0750	0.7220 ± 0.0943
YOLO11n	0.7403 ± 0.0599	0.6786 ± 0.0704	0.7727 ± 0.0956
YOLO26n	0.6738 ± 0.0753	0.6081 ± 0.0829	0.7378 ± 0.0935
YOLOv5s	0.7095 ± 0.0661	0.6513 ± 0.0741	0.7393 ± 0.0941
YOLOv8s	0.7045 ± 0.0623	0.6421 ± 0.0694	0.7434 ± 0.0878
YOLO11s	0.7368 ± 0.0594	0.6839 ± 0.0668	0.7450 ± 0.0792
YOLO26s	0.7267 ± 0.0678	0.6493 ± 0.0658	0.7623 ± 0.0884

**Table 3:** Performance comparison of single-modality and curriculum learning scenarios based on 100-seed repeated experiments.

Curriculum	mAP@50	Precision	Recall	Epochs
B-mode	0.7293 ± 0.0622	0.7538 ± 0.0932	0.6722 ± 0.0692	136.73 ± 48.27
Envelope	0.7162 ± 0.0653	0.7431 ± 0.0866	0.6618 ± 0.0798	125.41 ± 40.66
Phase	0.6347 ± 0.0667	0.7055 ± 0.1037	0.5867 ± 0.0807	126.73 ± 36.61
B-E-P	0.6562 ± 0.0635	0.7255 ± 0.0974	0.5926 ± 0.0740	115.23 ± 44.65
B-P-E	0.7392 ± 0.0604	0.7765 ± 0.0825	0.5706 ± 0.0729	104.62 ± 45.34
E-B-P	0.6471 ± 0.0728	0.7080 ± 0.0887	0.5984 ± 0.0849	106.41 ± 44.72
E-P-B	0.7432 ± 0.0611	0.7745 ± 0.0773	0.6730 ± 0.0746	114.70 ± 46.56
P-B-E	0.7402 ± 0.0640	0.7719 ± 0.0901	0.6784 ± 0.0730	103.03 ± 40.14
P-E-B (Proposed)	0.7445 ± 0.0625	0.7803 ± 0.0964	0.6842 ± 0.0709	116.00 ± 46.79

**Table 4:** Pairwise statistical comparisons between P-E-B and selected training scenarios based on repeated seed-wise results.

Metric	Comparison	Mean Difference (95% CI)	*p*-Value (Paired t/Wilcoxon)	Interpretation
mAP@50	P-E-B vs. B-mode	0.0145 [0.0040, 0.0249]	0.0064/0.0217	Significant improvement
mAP@50	P-E-B vs. E-P-B	0.0046 [−0.0044, 0.0137]	0.3212/0.2631	Not significant
mAP@50	P-E-B vs. P-B-E	0.0088 [0.0000, 0.0180]	0.0526/0.1145	Borderline tendency
mAP@50	P-E-B vs. B-P-E	0.0068 [−0.0026, 0.0163]	0.1739/0.1118	Not significant
Recall	P-E-B vs. B-mode	0.0178 [0.0028, 0.0320]	0.0170/0.0225	Significant improvement
Recall	P-E-B vs. E-P-B	0.0070 [−0.0078, 0.0216]	0.3558/0.2999	Not significant
Recall	P-E-B vs. P-B-E	0.0103 [−0.0034, 0.0240]	0.1427/0.1403	Not significant
Recall	P-E-B vs. B-P-E	0.0096 [−0.0059, 0.0249]	0.2228/0.1979	Not significant

## Data Availability

The public dataset used in this study is the Open Access Series of Breast Ultrasonic Data (OASBUD). No new clinical dataset was collected in this study. The experimental protocol, including the training orders, hardware environment, and software information, is described in the Materials and Methods section and Table 1. The original contributions and experimental results presented in this study are included in the article. Further inquiries can be directed to the corresponding author(s).
